# Reviewing Performance Measures of the Die-Sinking Electrical Discharge Machining Process: Challenges and Future Scopes

**DOI:** 10.3390/nano12030384

**Published:** 2022-01-25

**Authors:** Renu Kiran Shastri, Chinmaya Prasad Mohanty, Sitaram Dash, Karthick Muthaiah Palaniappan Gopal, A. Raja Annamalai, Chun-Ping Jen

**Affiliations:** 1 School of Mechanical Engineering, Vellore Institute of Technology, Vellore 632014, India; renukashastri2@gmail.com (R.K.S.); chinmaymohantymech@gmail.com (C.P.M.); sitaram.dash@vit.ac.in (S.D.); karthickmpg@gmail.com (K.M.P.G.); 2 School of Mechanical and Civil Engineering, MIT Academy of Engineering, Alandi, Pune 412105, India; 3 Centre for Innovative Manufacturing Research, Vellore Institute of Technology, Vellore 632014, India; 4 School of Dentistry, College of Dental Medicine, Kaohsiung Medical University, Kaohsiung 80708, Taiwan; 5 Department of Mechanical Engineering and Advanced Institute of Manufacturing for High-Tech Innovations, National Chung Cheng University, Chia-Yi 62102, Taiwan

**Keywords:** EDM, surface integrity, microhardness, surface roughness, recast layer, energy consumption

## Abstract

The most well-known and widely used non-traditional manufacturing method is electrical discharge machining (EDM). It is well-known for its ability to cut rigid materials and high-temperature alloys that are difficult to machine with traditional methods. The significant challenges encountered in EDM are high tool wear rate, low material removal rate, and high surface roughness caused by the continuous electric spark generated between the tool and the workpiece. Researchers have reported using a variety of approaches to overcome this challenge, such as combining the die-sinking EDM process with cryogenic treatment, cryogenic cooling, powder-mixed processing, ultrasonic assistance, and other methods. This paper examines the results of these association techniques on various performance measures, such as material removal rate (MRR), tool wear rate (TWR), surface roughness, surface integrity, and recast layer formed during machining, and identifies potential gap areas and proposes a solution. The manuscript is useful for improving performance and introducing new resolutions to the field of EDM machining.

## 1. Introduction

### The Electrical Discharge Machining Principle

Electrical discharge machining (EDM) is a thermal erosion process in which a controlled electric spark discharge occurs between the tool and the workpiece. As this process produces an eroding effect on the workpiece, a replica of the tool form is created on it. An electrical discharge phenomenon causes erosion between the tool and the workpiece. There is no mechanical contact between the two electrodes throughout the operation. Since the electrical conductivity of the tool and workpiece is a requirement for this process [1,2], the electrical resistivity of both electrodes must be between 100 and 300 Ωcm. In a dielectric medium, usually liquid, spark plasma is induced across inter-electrode gaps [3,4,5,6]. Dielectrics should have a high breakdown strength, quick recovery after a breakdown event, low viscosity, effective quenching/cooling, and flushing capability [7,8,9,10,11].

**Figure 1:**
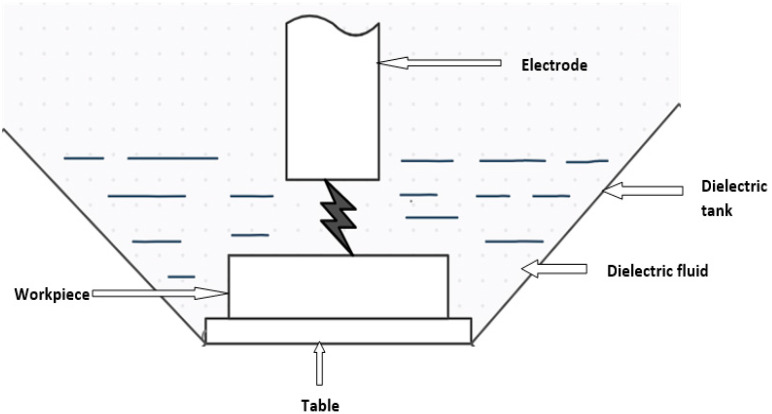
Mechanism of die-sinking EDM.

As the tool electrode approaches the workpiece, dielectric breakdown occurs, resulting in the formation of a plasma channel [12]. When a spark fails, the voltage drops and the current skyrockets. Since ionization of the dielectric medium present in the conductivity gap has created a plasma channel, the applied current produces heat, generating around a temperature of 8000 to 20,000 °C at the crater spot [13,14,15]. The size of a crater is determined by discharge energy, which can be controlled at the machine by adjusting the discharge current and duration [16,17,18,19]. The mechanism of die-sinking EDM is depicted in Figure 1. As the workpiece material and electrode heat up quickly, a molten metal pool forms at the workpiece’s surface [20,21]. A very small amount of metal is frequently evaporated in an instant. A portion of the debris is flushed out with dielectric medium, while the remainder resolidifies as a recast layer. The material removal rate is determined by the crater size and frequency of crater production, i.e., the discharge energy and frequency of discharges—the depth and craters determine the roughness of the machined surface. EDM is economically feasible due to its higher material removal rate with good accuracy and surface finish, which is achieved by designing a variety of spark generators and thus increasing productivity for various industrial applications. Various scholars have conducted a substantial amount of theoretical research on this topic [22,23,24,25].

**Figure 2:**
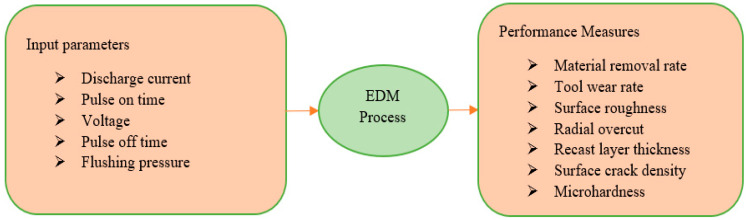
Performance measures of the EDM process with input parameters.

The study of the effect of process parameters on performance measures, such as material removal rate (MRR), tool wear rate (TWR), surface roughness (SR), and radial overcut, is critical in EDM. Different input parameters include discharge current, voltage, pulse on-time, pulse off-time, duty factor, and flushing pressure. All of these variables are critical during the machining process. The influence of discharge current, voltage, pulse on-time, pulse off-time, duty factor, and flushing pressure on performance measures has been extensively researched. According to leading researchers’ literature reports, pulse on-time [26,27,28,29,30,31,32,33], pulse off-time [26,27,28,30,32], voltage [26,28,33], current [30,31,32,33,34], and liquid flushing pressure [33,35] are the critical processes parameters that merit further investigation. Along with process parameters, the critical responses of the process, such as MRR, TWR, machined surface quality, and drilled hole accuracy, are used to evaluate EDM performance. In this context, a detailed review paper focusing on the performance measures of various advanced and hybrid EDM operations is presented. The paper also identifies numerous gaps, areas for future research, and solutions to improve the modern EDM process. Figure 2 depicts a summary of EDM process performance measures with input parameters.

## 2. Performance Measures

### 2.1. Material Removal Rate (MRR)

The MRR is an important performance measure in EDM because it is used to benchmark machining processes from the industry’s perspective. The MRR of the material should be as high as possible based on industry demand. Various materials, such as nickel and titanium, are used in the production of aircraft, military vehicles, and gas turbine components. Some of the materials used in this context are difficult to machine using conventional techniques. As a result, non-traditional machining plays an important role in the machining of such complex materials. These materials should be easily machined with EDM, and the MRR must also be determined. The MRR is the weight difference between the workpiece before and after machining divided by the machining time. The MRR has been reported by researchers. Kuppan et al. [36] reported the effect of process parameters on the MRR and surface roughness of Inconel 718. Inconel 718 has applications in the aerospace industry. Therefore, the machinability of this material is assumed significant. The study reported that the MRR of this material increases from 5.533 to 46.88 mg/min with peak current, irrespective of the pulse duration. Peak current is the most significant parameter related to the MRR of Inconel 718. Mohanty et al. [37] investigated the machinability of the Inconel 718 superalloy in EDM. The study showed that the MRR decreases from 11.22 to 4.45 mm^3^/min for brass, 25.54 to 12.38 mm^3^/min for copper, and 31.75 to 30.17 mm^3^/min for graphite electrodes, with an increase with open-circuit voltage. A machinability investigation on Inconel 718 was carried out on EDM by Kuppan et al. [38]. Mohan et al. [39] reported the EDM of the SiC/6025 Al composite. The study showed that the electrode produces a higher MRR (55 mm^3^/min) with a positive polarity electrode than a negative. As the volume percentage of SiC increases in the composite, the associated MRR exhibits a concomitant decline.

Pradhan et al. [40] used micro-EDM for machining of a titanium-based superalloy. In the machining of the titanium alloy, the MRR increases rapidly from 0.0062 to 0.033 mg/min with a pulse on-time. Yan et al. [41] used rotary EDM to cut the Al_2_O_3_/6061 Al composite. This study investigated the peak current and volume fraction of Al_2_O_3_ that have a significant effect on the MRR. Majumder et al. [42] used EDM to machine AISI 316LN stainless steel with a copper electrode. Mohanty et al. [43] reported that tool material, pulse on-time, and discharge current significantly affected machinability characteristics of Inconel 718 during the die-sinking EDM process. The study reported that the MRR increases from 20.05 to 48.9 mm^3^/min with the current when machined with a graphite electrode. Kapoor et al. [44] reported that the electrical conductivity of the brass wire is improved by cryogenic treatment during the wire EDM process. Gill et al. [45] investigated the machinability of a titanium alloy (Ti 6246) in an EDM-based drilling process when the alloy undergoes deep cryogenic treatment. Most of the researchers reported that cryogenic treatment invariably improves the MRR [46,47,48,49,50,51,52].

Most researchers reported powder-mixed EDM to improve the rate of EDM-based machining (PMEDM). Powder-mixed EDM (PMEDM) is an enhanced EDM technology in which the dielectric medium is mixed with a fine, abrasive, electrically conductive powder. Metallic powders suspended in the dielectric medium reduce their insulating strength, increasing the inter-electrode gap conditions, which improves EDM performance and results in a better surface finish than conventional EDM. Tall et al. [53] reported machining of the Al/Al_2_O_3_ metal matrix composite with the EDM process using aluminum powder (average size of 15 µm) in the kerosene dielectric medium. They found that the addition of aluminum powder in the dielectric medium improves the MRR. Kolli et al. [54] reported that the MRR is improved when graphite powder (14 g/L) and surfactant (varied between 0.25 and 15.0 g/L) are added to the dielectric fluid during EDM of the titanium alloy. Kansal et al. [55] used silicon powder (average particle size 30 µm) in a dielectric fluid to improve the machining rate of die steel. The study reported an increase in machining rate from 2.67 to 4.58 mm^3^/min at a 3 g/L silicon powder concentration. Singh et al. [56] studied the abrasive mixed PMEDM and reported the highest MRR (0.57 g/min) at the concentration of SiC of 8 g/L. Kumar et al. [57] reported the peak current, powder concentration, and pulse duration. These were considered as influencing parameters during the PMEDM of the Al-SiC_P_ metal matrix composite. The material contained 10% SiC particles (by volume) with an average particle size of 25 µm as reinforcement. The study reported a higher MRR, that is 2.93 mm^3^/min, at a silicon powder concentration of 4 g/L. According to the PMEDM studies, increasing the powder concentration in dielectric fluid improves machining performance [58,59,60,61,62,63].

Some studies on ultrasonic-assisted EDM claimed that by applying ultrasonic vibrations to the electrode, debris would be removed from the machining area via a high-frequency pumping action. Ultrasonic vibration (UV)-aided EDM is a hybrid method in which ultrasonic vibration is incorporated into the EDM process. Ultrasonic vibration (at a frequency of 20 kHz or higher) is used during the EDM process to improve the process’s flushing efficiency. Depending on the applications and challenges encountered during the EDM process, ultrasonic vibration can be used on the tool, workpiece, or even dielectric medium. Kremer et al. [64] investigated how ultrasonic vibrations affected EDM performance. The application of ultrasonic vibrations to the electrode improves the flushing action, resulting in an increase in MRR. Abdullah et al. [65] reported an improvement in the MRR from 0.018 to 0.0145 mm^3^/min using the ultrasonically vibrated tool during EDM of cemented tungsten carbide. Ultrasonic vibrations produce a higher number of discharges, and due to this, the MRR is enhanced. Lin et al. [66] investigated machining characteristics of Ti-6Al-4V using an ultrasonic approach coupled to EDM. The study found that when EDM and USM are combined, the MRR (0.087 to 1.42 mm^3^/min) improves due to enhanced discharge. Hence, several researchers combined the ultrasonic machining approach with EDM to enhance machining performance and efficiently improve the MRR [67,68,69,70,71,72,73,74,75,76,77,78].

### 2.2. Tool Wear Rate (TWR)

The TWR is the amount of material lost from the electrode during the machining process. It is calculated by dividing the difference in electrode weight before and after machining by the time spent machining. TWR stands for the time rate of material loss. It is a critical performance measure in the industry because it affects cost and productivity. The amount of material that erodes during the EDM process is determined by the material of the electrode and the machining conditions. As a result, during the EDM process, researchers focused not only on the MRR but also on the TWR. Researchers have investigated various electrode materials and machining conditions to optimize electrode wear during the EDM process. Electrode wear also depends on the thermal conductivity of the electrode material. As the electrode has high thermal conductivity, heat is quickly dissipated through the electrode, resulting in reduced wear. Straka et al. [79] used copper electrodes to machine tool steel by EDM. The copper electrode was chosen because of its high thermal conductivity, which reduces the TWR. The impact of peak current, pulse off-time, voltage, and pulse on-time on the TWR was studied. The study reported an increase in the TWR from 0.3 to 360 µm^3^/min with peak current. Amorim et al. [80] used die-sinking EDM to machine tool steel with copper and graphite electrodes. The study showed that these two electrodes produced similar volumetric relative wear for the positive polarity and relatively low wears rates (that is, 30%) for negative polarity. Khan et al. [81] reported the machining of aluminum and mild steel employing copper and brass electrodes. The lowest electrode wear ratio (0.012) was reported while machining aluminum with the copper electrode.

Zarepour et al. [82] have carried out a statistical analysis of electrode wear in EDM. The copper electrode has been used to cut the DIN 1.2714 tool steel, which is used to fabricate mandrels and forging dies. The electrode wear ratio increased up to 0.57% with an increase in current values. Wang et al. [83] formed a semiempirical model on workpiece material removal and tool wear. Khan et al. [84] reported the performance of aluminum and copper electrodes in the EDM process. As reported in their study, copper electrodes exhibited much less wear (approximately 1.8 g) than aluminum during the EDM process carried out on stainless steel.

Some researchers have proposed composite electrodes for the EDM process. These researchers have proposed a novel electrode material based on a copper and TiB_2_ composite. Before introducing Rapid Prototyping technology, sintering was used to establish bonding between copper and TiB_2_ [85]. Puertas et al. [86] proposed machining a ceramic compound based on tungsten carbide with the copper electrode for industrial applications. Kunieda et al. [87] reported spectroscopic measurements of the vapor density of the electrode material for the determination of the electrode wear ratio. Mascaraque-Ramrez et al. [88] investigated tool degradation at the electrode workpiece interface, focusing on the central and border zones of the active electrode area. Additional investigations on electrode wear have been reported by the majority of the researchers [89,90,91,92,93,94,95,96,97,98].

**Figure 3:**
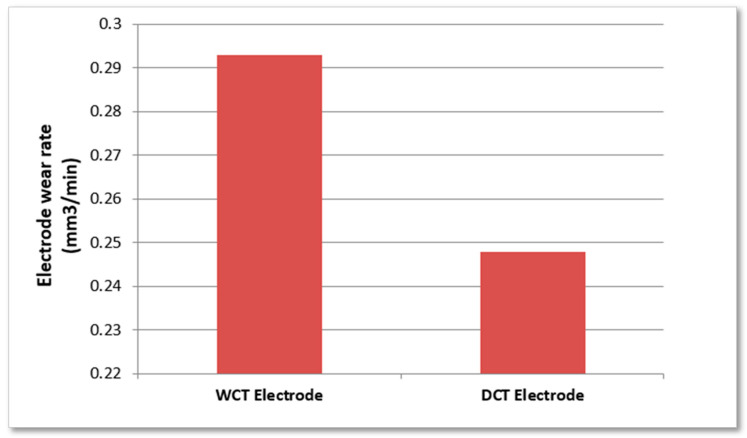
Electrode wear rate of the copper-tungsten electrode without cryogenic treatment (WCT) and with deep cryogenic treatment (DCT) during EDM of the Ti-5Al-2.5Sn alloy [101].

According to the literature review, prior cryogenic treatment reduces tool wear because it increases the material’s electrical conductivity. Kumar et al. [99] proposed that cryogenically cooled electrodes have less tool wear than standard EDM electrodes. Kanth et al. [100] investigated the tool wear rate of cryogenically treated tool electrodes such as graphite, copper, and brass. According to the study, deep cryogenic treatment reduces tool wear when compared to non-cryogenic tools. The electrode wear rate for a copper-tungsten electrode with and without cryogenic treatment while EDM of the Ti-5Al-2.5Sn alloy is reported by Kumar et al. [101]. Figure 3 shows the Electrode wear rate of the copper-tungsten electrode without cryogenic treatment (WCT) and with deep cryogenic treatment (DCT) during EDM of the Ti-5Al-2.5Sn alloy. Several studies [102,103] have found that cryogenic treatment improves the TWR compared to untreated tools.

Da Silva et al. [104] reported that cryogenically treated tools have performed better in comparison with the untreated ones in the Brandsma rapid facing test. In some cutting situations, the difference was as high as 44%. Kumar et al. [105] utilized cryogenically treated and untreated copper electrodes to machine Inconel 718 in PMEDM. This research was conducted to find the machining efficiency concerning the TWR. This study reported improvement in the TWR by using a cryogenically treated tool. Cryogenic treatment improves the electrode material’s hardness, wear resistance, and thermal and electrical properties. Kumar et al. [106] also proposed a TWR model based on PMEDM for cryogenically treated electrodes. Sundaram et al. [107] investigated the electrode wear ratio (EWR) of the copper electrodes. Two different treatment methods, namely, deep cryogenic treatment and typical standard cold treatment, were adopted. The electrode wear ratio was lowered from 20.33% to 19.58% and 19.78%, for cold treatment and deep cryogenic treatment, respectively.

### 2.3. Surface Roughness

In EDM, the surface is eroded by spark plasma-induced material ejection. The spark erosion process causes micro-voids and crater formation on the machined surface. Therefore, erosion-induced surface roughness needs to be determined. Several studies have been reported on the measurement of the roughness of the EDM machined surface. Guu et al. [108] proposed the study of EDM processing of AISI D2 tool steel. Surface roughness was studied using a profilometer, which illustrates the variation of surface roughness with pulse current from 1.3 to 11.0 µm. Rahul et al. [109] investigated surface characteristics of Inconel 718 in EDM. As peak current increases, spark plasma density and energy rise. An increase in peak current causes increases in surface roughness from 3.8 to 10.33 µm. Bhattacharyya et al. [110] reported the effect of EDM process parameters on the surface roughness of die steel. It shows a variation of surface roughness from 2.4 to 5.08 µm with the current. Keskin et al. [111] studied the influence of EDM process parameters on the surface roughness of steel workpieces. The study found that as discharge duration increased, surface roughness increased. Lee et al. [112] investigated the effect of key input parameters on the hole enlargement, white layer thickness, and surface roughness of AISI-1045 and AISI-D2 workpieces in EDM. Liao et al. [113] proposed a modified circuit for achieving a good surface finish on EDM wire-cut surfaces. The effect of each major element on surface roughness was evaluated and ideal values for all parameters were obtained, resulting in a fine surface with a roughness of Ra = 0.22 µm. Mandal et al. [114] developed two post-processing procedures in wire EDM: grinding and etching-grinding, to increase the surface integrity of the machined surface. The proposed post-processing technique was shown to be extremely effective in producing a surface with an average roughness of less than 0.024 µm. Aspinwall et al. [115] used wire EDM to cut Inconel 718 and Ti-6Al-4V alloys. The surface roughness and integrity of the machined surface were reported in the study. The surface roughness varies from 0.21 to 2.36 µm for Ti-6Al-4V and 0.21 to 2.93 µm for Inconel 718 during machining. Bleys et al. [116] investigated the effect of EDM on the quality of machined tool steel and mold surfaces. Goyal et al. [117] investigated the effect of powder metallurgy electrodes such as copper-manganese (70:30) and copper-manganese (80:20) on EDM-processed surfaces. The copper-manganese (70:30) combination has the greatest surface roughness value of 12.37 Ra. Other researchers have also reported on their research into surface roughness [118,119,120,121,122,123].

### 2.4. Surface Integrity

**Figure 4:**
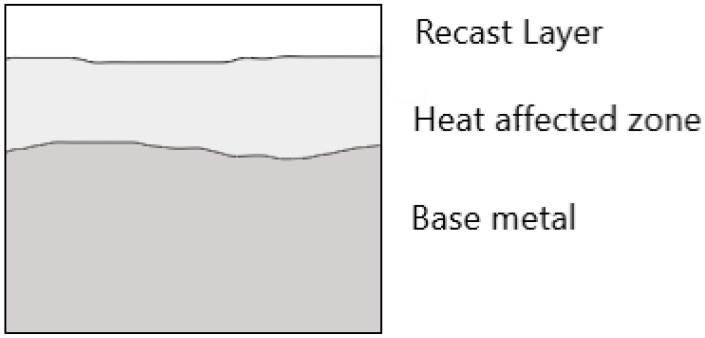
Evolution of layered zones in an EDM-processed specimen.

Some molten material resolidifies on the workpiece surface because the dielectric fluid cannot remove all of the molten material from the cutting region during EDM. As shown in Figure 4, the resolidified material produced a distinct layer on the machined surface, known as a recast layer.

**Figure 5:**
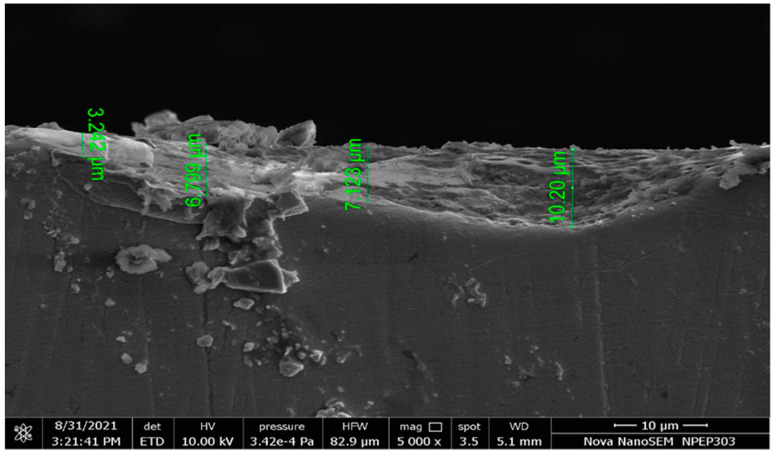
SEM image of Recast layer thickness (RCT) for the Nimonic C-263 workpiece machined with a copper electrode.

Craters, pockmarks, white or recast layers, and cracks are generated on the workpiece’s surface during EDM machining and decrease the surface finish. Figure 5 shows an SEM image of the machined surface representing recast layer thickness.

#### 2.4.1. RCT on Workpiece

Rahul et al. [99] looked into the surface integrity of Inconel 825 machined with cryogenically treated copper electrodes. Cracks on the surface of the workpiece are smaller when machined with cryogenically treated electrodes, according to the study. The thickness of the recast layer was also found to be greater (approximately 26%) in the case of a cryogenically treated electrode compared to non-cryogenically treated electrodes.

**Figure 6:**
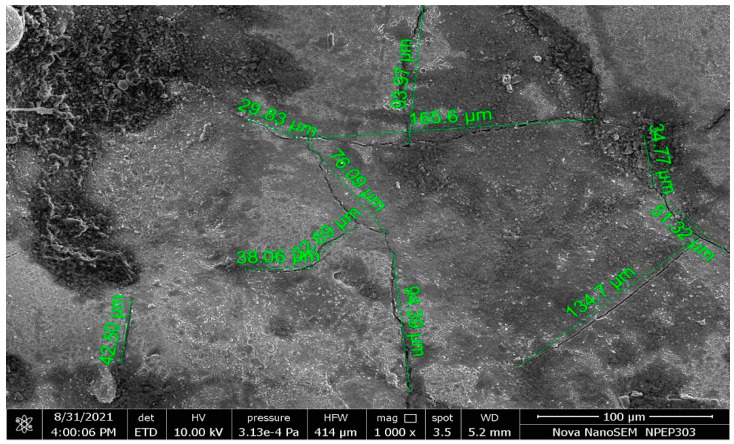
SEM image of the machined surface showing surface cracks, spherical drops, and crack length.

Guu et al. [108] published their findings on the EDM machining of AISI D2 tool steel. The thickness of the recast layer was measured in the study. The study also discovered that as pulse duration and current increased, the recast layer thickness rose up to 36 µm. Rahul et al. [109] investigated the surface integrity of Inconel 718 and measured surface cracks to assess crack severity. Surface cracks, debris, and globules are visible on the machined surface. According to the study, the thickness of the white layer increases from 19.074 to 20.308 µm with pulse duration. Bhattacharya et al. [110] investigated the thickness of the white layer, surface roughness, and surface crack density of die steel in EDM. According to the study, the peak current should be low in order to reduce the thickness of the white layer—the medium value and minimum pulse on-time are recommended. Lee et al. [112] reported that the pulse current and pulse duration are significant factors for white layer thickness. Figure 6 shows the SEM image of the machined surface. The image indicates the presence of surface cracks and spherical drops on the machined surface. The surface crack lengths are demarcated on the image. These are used to calculate the surface crack density.

Mandal et al. [114] used WEDM to machine a Nimonic C-263 alloy. To remove the recast layer, the study proposed two post-processing techniques: grinding and etching-grinding. As a result, the surface integrity of the machined surface was improved. Aspinwall et al. [115] investigated surface integrity after EDM machining of Ti-6Al-4V and Inconel 718 alloys. They have carried out multiple trim-cut strategies to employ minimum surface damage. The study found that no recast layer forms after multiple trim cuts. Bley’s et al. [116] discussed the influence of EDM machining on the quality of surface and sub-surface layers. Li et al. [118] proposed wire- and die-sinking EDM for machining an Inconel alloy. Since Inconel has a higher toughness, the study found that it has a lower crack density. In this direction of surface integrity, few studies were reported [124,125,126,127,128].

#### 2.4.2. RCT on Electrode

Along with these studies, some researchers explored the surface integrity of electrodes during EDM. Kumar et al. [101] used cryogenically treated copper-tungsten electrodes to machine a titanium alloy with EDM. They reported that the electrode surface develops various defects due to the recast layer formation on the electrode surface. Kumar et al. [106] reported machining of three grades of titanium alloy with powder-mixed EDM and observed an increase in holes, pockmarks, debris, etc., on the tool surface with an increase in current.

Table 1 provides a summary of the novelties available in the literature for improving the performance of EDM.

**Figure 7:**
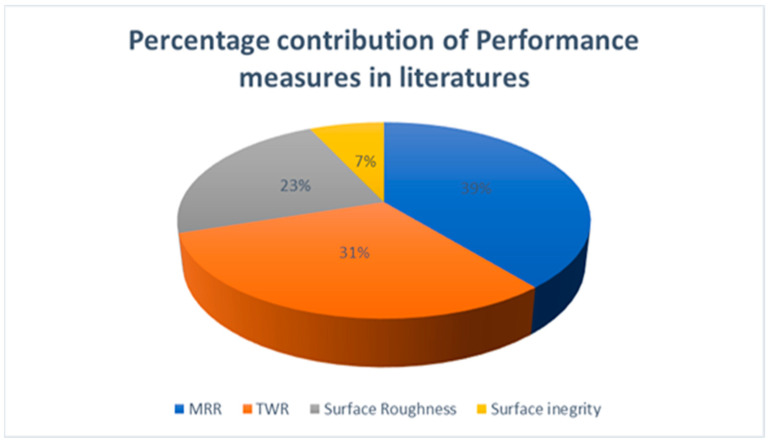
Percentage contribution of performance measures in the literatures.

Figure 7 shows the percentage contribution of MRR, TWR, SR, and surface integrity studied in various works. In EDM, it can be seen that 39% of research publications focus on MRR, and 31% contribute to TWR. It can be seen that 23% of research work is carried out on SR, and 7% of research work is carried out on surface integrity in EDM. From the chart, researchers have primarily focused on performance measures such as MRR, TWR, and surface roughness of the machined parts. Surface integrity, surface crack density, radial overcut, and microhardness of machined parts have not received sufficient attention. Since EDM is a thermal process and is commonly used in the machining of hard materials, which are widely used in a variety of industrial sectors, the heat generated during machining can have a significant impact on the machined surface quality and the properties of the workpiece. As a result, an attempt must be made to comprehend and analyze the EDM process attributes on the surface crack density, radial overcut, and microhardness of machined parts.

## 3. Statistical Tools and Artificial Intelligence Techniques Applied in EDM

**Figure 8:**
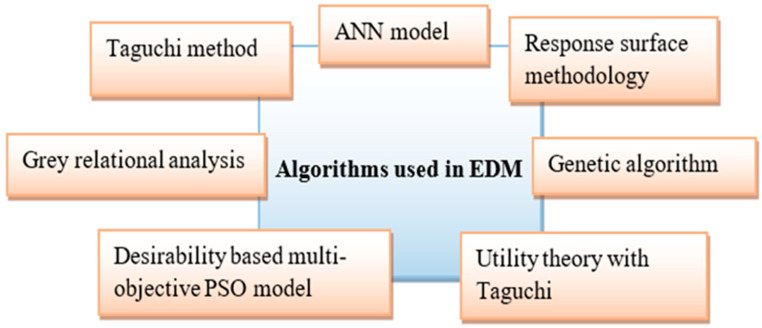
Statistical Tools and Artificial Intelligence Techniques applied in EDM.

Figure 8 demonstrates the statistical tools and artificial intelligence techniques which are used for processing in EDM. 

Most of the researchers implemented the Taguchi method for analysis [27,37,40,44,54,55,57,82,83,105,106,112,113]. Some researchers used the response surface methodology (RSM) technique for analyzing the EDM process parameters [28,30,31,32,33,36,38,110,114]. The artificial neural network (ANN) model is implemented in some research work for finding optimum parameters in EDM [34]. The genetic algorithm model is adopted in [39]. In [42], desirability-based multi-objective particle swarm optimization (DMPSO) is used for EDM processing. A semiempirical model was developed based on machining parameters in [53]. 

According to reports, the essential factors of EDM are peak current, pulse duration, voltage, pulse off-time, and so on. It is critical to select optimal parameters when machining. These parameters have a direct impact on the component’s MRR, TWR, surface roughness, and so on. As a result, determining the best EDM parameters is critical. Several optimization techniques have been reported in the literature, and a few of them are highlighted in Table 2. The quantum behaved particle swarm optimization (QPSO) and PSO have been employed in [37] for optimization. The desirability function approach has been used in [30,38]. PSO is used for optimization in [43]. The principal component analysis-based grey technique (Grey-PCA) has been adopted in [53]. Most papers used grey relational analysis (GRA) for finding the optimum parameters in their work [56,122]. Further, the ANN integrated non-dominated sorting genetic algorithm II (NSGA-II) is employed in [129]. A genetic algorithm is used for optimization in [130]. Energy efficiency optimization was carried out with the help of NSGA-II optimization in [131]. The improved PSO, called the multi-objective particle swarm optimization (MOPSO) technique, was used in [33,132]. Utility theory with the Taguchi method is used in [107,133].

## 4. Conclusions

The present manuscript attempted to review the literature in the EDM-based fabrication process and identify gap areas, for further investigations. A critical assessment of the operational efficiency of the EDM process has been carried out for various materials and electrodes in terms of MRR, TWR, surface roughness, and crack density. EDM has substantially enhanced the standard of machining operations in recent years. A summary of research trends in ultrasonic vibration EDM, EDM in different water-based dielectric fluids, EDM with powder compounds, dry EDM, and several modeling techniques for forecasting EDM performances was presented. Researchers have recently used various types of vegetable oils as a dielectric medium during EDM and discovered that they had a higher MRR, a better surface quality, and emit fewer hazardous gases than hydrocarbon-based dielectric fluids. The researchers also concentrated their efforts on the micro-EDM and its diverse uses. EDM is also being studied for hard-to-machine materials such as metal matrix composites, high-hardness steel, and superalloys.

**Figure 9:**
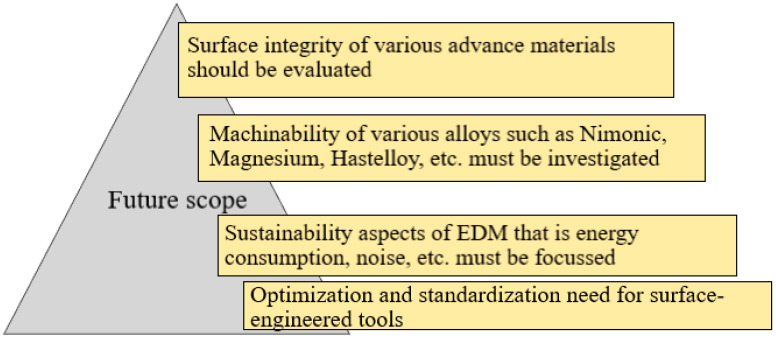
Future scope of EDM.

The survey revealed technical gaps concerning benchmarking MRR, TWR, and other machining parameters for novel electrode materials. Gap areas exist regarding introducing novel processes based on PMEDM, ultrasonic cavitation assistance, and cryogenic treatment. Standardization and optimization requirements of the above area concerning MRR and TWR are yet to advance. The level of scientific and technical understanding in some of the above areas remains sparse. The synergy between the concurrent introduction of abrasive particles and directed spark plasma jet also requires vigorous investigation. Figure 9 depicts the future scope of EDM.

The critical gap areas and suggestions made to enhance the EDM performance are as follows.

1. Surface integrity, surface crack density, microhardness, and radial overcut of the machined surface have not been given adequate importance, as measured by the researchers. Therefore, an attempt must be made to evaluate these measures with the greatest priority.
2. It was also observed that a few works have been carried out on aerospace materials such as Nimonic, Titanium alloys, Hastelloy, Hayeness alloys, Magnesium alloys, etc. [27,114,119,122,134]. These alloys have vital applications in manufacturing parts in the aircraft and automotive industries. Hence, the machinability of these novel workpieces must be explored in EDM.
3. Few studies have looked at the process’s long-term viability, such as energy consumption, machining noise, and machining debris during EDM machining.
4. In order to produce energy-efficient and clean EDM operations, these sustainable measures, as well as other machining measures, should be evaluated. Another example is the prior cryogenic treatment of the EDM tool to reduce the TWR. Despite research demonstrating the method’s efficacy, no standardization of the procedure or refusal to establish a cutting process for hard materials has been proposed. A similar gap exists when it comes to the use of powder-mixed composites and the accompanying ultrasonic assistance.
5. Although not discussed, the thermal model of heat transfer efficiency and flushing characteristics needs to be addressed for novel ultra-hard material processing. In addition, there is an optimization and standardization need for surface-engineered tools.
6. There are several reports in the literature for achieving low surface roughness in the EDM process. PMEDM (powder-mixed EDM) has been attempted for machining rigid material with the desired accuracy. However, quantitative correlation and benchmarking of process parameters remain to be implemented.
7. The survey revealed a considerable scope for further research. The discharge dynamics and heat transfer characteristic thermal modeling, flushing out effects, and assistance from ultrasonic cavitation need quantitative understanding. Enhanced MRR and reduced TWR are critical essential assessment criteria for an EDM process. Potential benefits from the use of surface-engineered tools also need further probing.

## Figures and Tables

**Table 1:** Major findings available in the literature to improve the performance of EDM machining during the process.

Year	Author	Novelties in EDM
2004	Singh et al. [12]	EDM was utilized to machine hardened tool steel with different tool electrodes such as copper, copper-tungsten, brass, and aluminium.
2007	Khanra et al. [29]	The ZrB_2_-Cu composite electrode was used to machine the material in EDM. High MRR and low TWR were reported by using this composite electrode.
2007	Abdullah and Shabgard [65]	The effect of ultrasonic vibration-assisted copper tools on the machining of cemented tungsten carbide was investigated.
2010	Abdulkareem et al. [26]	Electrode cooling was carried out during EDM of titanium alloy. The effect of electrode cooling on electrode wear was deliberated.
2013	Gopalakannan et al. [28]	EDM was employed to machine a metal matrix nanocomposite synthesized by the ultrasonic cavitation method.
2015	Dewangan et al. [32]	A Grey-Fuzzy logic-based hybrid optimization technique was reported to improve the surface integrity of material during EDM processing.
2017	Kumar et al. [99]	Surface integrity and metallurgical characteristics of Inconel 825 were investigated by machining with cryogenically treated copper electrodes.

**Table 2:** Optimization analysis.

Optimization Technique	Citation
Desirability approach	[30]
MOPSO	[33,132]
QPSO	[37]
PSO	[43]
Desirability based PSO	[42]
Grey-PCA	[53]
GRA	[56,122]
Utility theory with Taguchi	[107,133]
ANN integrated NSGA-II	[129]
GA	[130]
NSGA-II	[131]

## Data Availability

No new data were created or analyzed in this study. Data sharing is not applicable to this article.
